# A Case of Rocuronium-Induced Anaphylaxis Managed With Sugammadex

**DOI:** 10.7759/cureus.81701

**Published:** 2025-04-04

**Authors:** Ahsum Khan, Kathleen Parr

**Affiliations:** 1 Anesthesiology, George Washington University School of Medicine and Health Sciences, Washington D.C., USA

**Keywords:** anesthesia induction, drug induced anaphylaxis, peri-operative anaphylaxis, rocuronium induced anaphylaxis, sugammadex

## Abstract

Anaphylaxis is a severe, life-threatening complication that can result in complete cardiovascular and respiratory collapse. Peri-operatively, this complication is most often seen after the intravenous (IV) induction of general anesthesia, and the most common responsible agents are non-depolarizing neuromuscular blocking agents. In this report, we describe a case of perioperative anaphylaxis induced by the non-depolarizing neuromuscular blocker rocuronium. Sugammadex, a reversal agent for the non-depolarizing muscle relaxants rocuronium and vecuronium, was administered shortly after induction. The patient showed rapid clinical improvement in blood pressure, heart rate, and oxygen saturation values within minutes of sugammadex administration. We suggest that sugammadex should be considered as an adjunct therapy when treating anaphylaxis caused by rocuronium.

## Introduction

Anaphylaxis is a rapidly developing, potentially fatal, severe allergic reaction. Common causes include, but are not limited to: latex, drugs, contrast dye, insects, and/or foods [1]. As an immunoglobulin E (IgE)-mediated type 1 hypersensitivity reaction, inflammatory mediators and cytokines (including histamine, tryptase, proteoglycans, interleukin (IL)-4, and IL-13) are key to the inflammatory cell response and antibody generation involved in this severe allergic reaction [1].

After exposure to a causative agent, patients can develop anaphylaxis within minutes to hours. Approximately 0.05-2% of the global population will experience anaphylaxis at least once in their lifetime. In patients undergoing general anesthesia, the frequency is between 1:5,000 and 1:20,000 cases [2,3].

Non-depolarizing muscle relaxants are the most common cause of anaphylaxis in patients receiving general anesthesia. The estimated frequency is 1:6,500 administrations [2]. The second most common cause in anesthesia is B-lactam antibiotics. Cefazolin causes anaphylaxis in 1:10,000 administrations [3]. Common signs and symptoms include urticarial rash, edema, lightheadedness, syncope, shortness of breath, wheezing, low blood pressure (BP), elevated heart rate (HR), diarrhea, and vomiting [2,4].

Once identified, initial treatment for anaphylaxis includes the administration of intravenous (IV) fluids and epinephrine, with antihistamines used as adjuncts. Patients with bronchospasms should be given nebulized beta-agonists. Monitoring patients for signs of respiratory distress is crucial, as airway compromise can quickly develop. With prompt recognition and treatment the prognosis with anaphylaxis is excellent, as 99.5% of cases fully resolve [1,2].

In this report, we describe the case of a 40-year-old male who developed perioperative anaphylaxis likely secondary to an induction dose of rocuronium. He was given standard treatment for perioperative anaphylaxis via the American Society of Anesthesiologists (ASA) guidelines [2]. He was also given a reversal dose of sugammadex for his neuromuscular blockade induced by rocuronium. It was thought that reversal would prevent further proliferation of his anaphylaxis by eliminating the antigenicity of the agent [2]. Shortly after the administration of sugammadex, the patient showed rapid clinical improvement. This report may help demonstrate a novel approach to the management of rocuronium-induced anaphylaxis. Sugammadex may be considered a useful adjunct to the current standard management of perioperative anaphylaxis secondary to rocuronium.

## Case presentation

Our patient was a 40-year-old male with a BMI of 24 presenting for a C4-C5 anterior cervical discectomy and fusion. His past medical history was significant for seasonal allergies and C4-C5 myelopathy. He had no known drug allergies. Preoperative examination and evaluation was unremarkable. No difficulty was anticipated with mask ventilation or endotracheal intubation.

The patient was brought into the operating room and given 2 mg of IV midazolam as an anxiolytic. American Society of Anesthesiologists (ASA) standard monitors were applied. Preinduction vitals were a blood pressure (BP) of 114/69 mmHg, heart rate (HR) of 87 bpm, and oxygen saturation (SpO2) of 100%. The patient was then preoxygenated via a facemask with 100% oxygen. During preoxygenation, 2 grams of cefazolin was pushed over 3 minutes. There were no signs or symptoms of allergic reaction at this time. General anesthesia was then induced via IV with 150 mg of propofol, 100 mg of lidocaine, 100 mcg of fentanyl, and 60 mg of rocuronium. Mask ventilation was not difficult, and the patient was easily intubated with a 7.5 mm endotracheal tube (ETT). Inhaled sevoflurane was started after intubation for maintenance of anesthesia.

Approximately five minutes after the induction of anesthesia, the patient was noted to be hypotensive with a BP of 77/42 mmHg. Initial treatment was 100 mcg phenylephrine IV. There was no significant BP response. Concurrently, the patient’s HR also increased to 130 bpm, and the SpO2 dropped to 89%. An additional dose of 300 mcg of phenylephrine was given, again with no significant BP response (BP 73/42).

At this time, the patient’s skin was examined, revealing a diffuse red rash on the chest and face. The patient’s tongue and lips had also started to swell. Respiratory wise, peak airway pressures were stable at 15 mmHg, and breath sounds were clear, equal, and bilateral. It was determined that the patient met the criteria for anaphylaxis. Epinephrine 10 mcg was given IV. The patient’s BP responded to 83/50 mmHg. Two additional 10 mcg epinephrine doses were then given, spaced approximately 2 minutes apart. The BP then increased to 85/54 mmHg. A 20G left radial arterial line was placed, and an 18G IV was placed in the right arm. An epinephrine infusion was started at 2 mcg/min through an in situ 18G IV in the left arm. Diphenhydramine 50 mg IV, famotidine 20 mg IV, and decadron 10 mg IV were also given.

**Figure 1:**
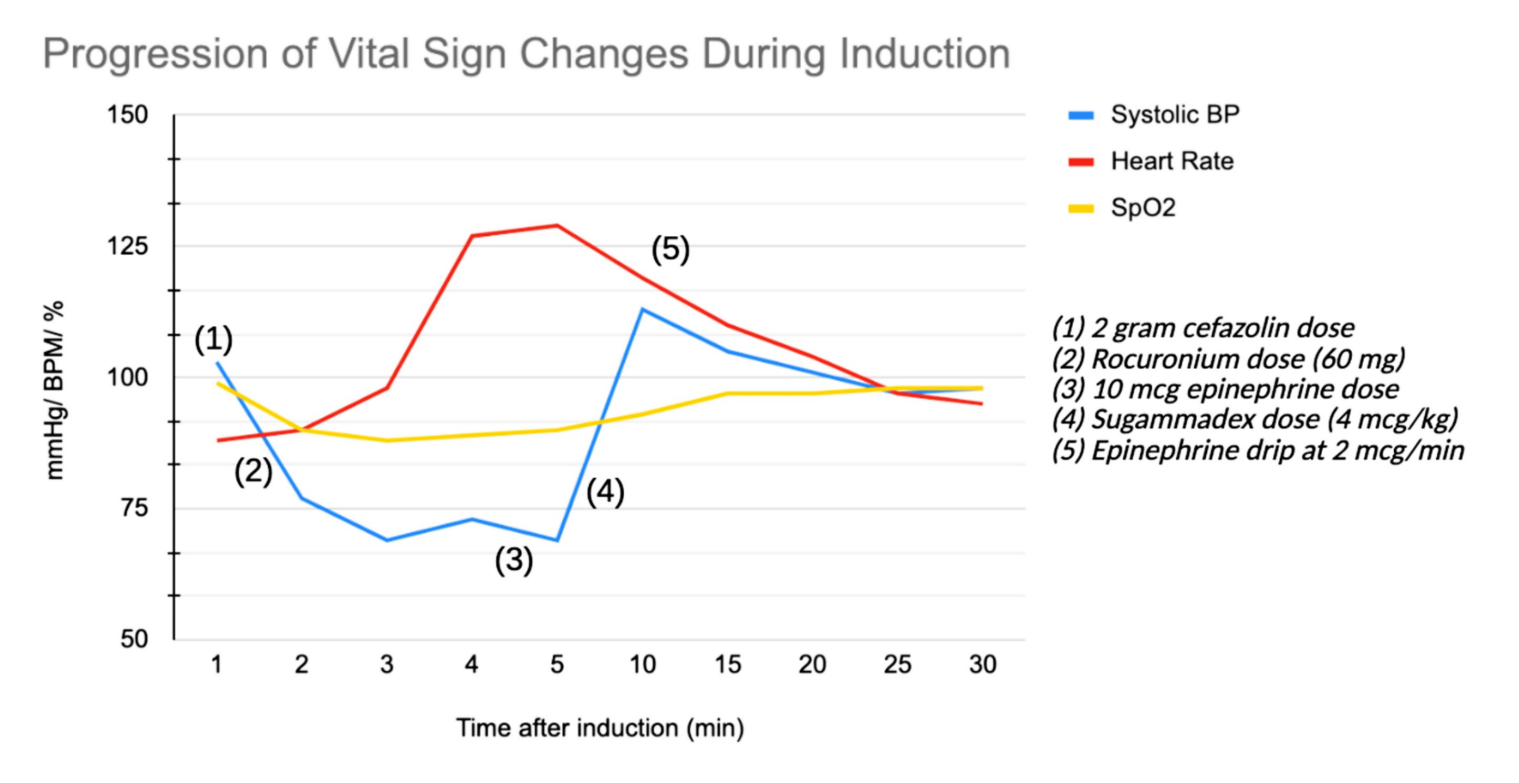
Progression of vital sign changes during induction

Despite these therapies, the patient's vital signs failed to show sustained improvement. Two minutes after the start of the epinephrine infusion, the BP was 87/42 mmHg, HR was 137 bpm, and SpO2 was 93%. As it was thought rocuronium was the probable cause of the anaphylactic reaction, the decision was made to administer sugammadex in an attempt to remove the offending agent from the circulation. Therefore, a 4 mcg/ kg dose was administered IV. Two minutes after sugammadex administration, the patient’s vitals had significantly improved: BP 113/65 mmHg, HR 120 bpm, and SpO2 97%. A progression of vital sign changes during induction is provided in Figure 1.

As the patient’s vitals were steadily improving, an open discussion took place between the anesthesia and surgical teams, and the parties felt comfortable proceeding with the case. No further neuromuscular blockade was administered. Vitals were stable for the remainder of the case on a 2 mcg/min dose of epinephrine. Bilateral breath sounds remained clear, and peak airway pressures never increased. At the end of the case (three hours later), the patient’s rash had improved in color and size, and the lip and tongue swelling had diminished. The epinephrine infusion was also titrated off. An arterial blood gas was obtained 10 minutes prior to the end of the case, which revealed a pH of 7.31, PCO2 of 45, PO2 of 213, and a HCO3 of 23, consistent with mild respiratory acidosis. The patient emerged from general anesthesia without incident, and the ETT was removed after careful testing for airway edema. The patient was taken to recovery in stable condition, on 2 L/min O2 via a nasal cannula, which was titrated off to 15 minutes later in the post-anesthesia care unit (PACU).

The patient was admitted to the hospital for 23 hours after surgery for observation. The patient remained clinically stable and was cleared for discharge on postoperative day one. It was recommended postoperatively that the patient follow up with an allergist as an outpatient for allergy testing. A list of medications given at induction was given to the patient. Unfortunately, we did not think to draw tryptase levels at the time of the reaction; however, it was clear that the patient was undergoing a severe allergic reaction.

## Discussion

Anaphylaxis can be generally classified into one of two types: anaphylactic shock and anaphylactoid reaction. Anaphylactic shock is a type 1 hypersensitivity reaction, which is IgE mediated [3]. It results in systemic vasodilation, which may result in a drop in BP <30% of baseline or a systolic pressure of <90 mmHg [2]. An anaphylactoid reaction is a non-immune reaction that does not involve IgE medication and is not a hypersensitivity reaction. It is due to direct mast cell degranulation. Anaphylaxis and anaphylactoid reactions are both treated similarly [2,3].

Muscle relaxants are the top cause of anaphylaxis in patients undergoing general anesthesia, followed by beta-lactam antibiotics, latex, local anesthetics, and opioids. A two-year study conducted in France by Raft found that 58.2% of perioperative anaphylactic reactions were caused by non-depolarizing muscle relaxants [4]. It is thought that their quaternary ammonium structure may trigger anaphylaxis in patients. This structure is found in various cosmetics, shampoos, soaps, and toothpastes [3]. After being sensitized to this structure via daily use, the initial exposure to a nondepolarizing muscle relaxant, such as rocuronium, can trigger anaphylaxis [4].

During general anesthesia, there are multiple opportunities for anaphylaxis to occur, but the most common time is at the induction of anesthesia. Over 90% of perioperative cases of anaphylaxis occur at induction [3]. As multiple drugs are typically given during induction, it can be difficult to determine which agent is the cause. All agents given before the reaction should be considered suspect and stopped. Determination of the likely causative agent should be based on incidence, reaction timing, patient presentation and history. In our case, rocuronium seemed the most likely agent based on these factors [2-4].

Hemodynamic collapse and respiratory failure are the top concerns in anaphylaxis. Signs of either or both of these require prompt and deliberate treatment. Signs of hemodynamic collapse should be treated with IV fluids and epinephrine. Signs of respiratory distress and bronchoconstriction should be treated with beta-agonist agents and epinephrine, and prompt airway securement via intubation should be considered [4,5].

In our case, the patient had cardiovascular and respiratory symptoms. The patient quickly developed hypotension, tachycardia, and hypoxemia. Multiple doses of phenylephrine and fluid boluses were ineffective in treating this patient’s hypotension and sustained tachycardia. Once a bright red rash was noticed on the patient’s anterior thorax and face, and it was noted that the patient’s lips and tongue were swollen, a presumptive diagnosis of an anaphylactic reaction was made. A 1 L lactated ringers (LR) fluid bolus was started, along with three 10 mcg IV epinephrine boluses, spaced two minutes apart. After mild improvement in BP and HR, an epinephrine infusion was started at 4 mcg/min. Rocuronium and cefazolin were considered to be the most likely causes of anaphylaxis. To remove rocuronium from the circulation and prevent further reaction, sugammadex was administered at 4 mcg/kg [5].

Previous reports have described sugammadex as a potential treatment for rocuronium-induced anaphylaxis. The theory is that as sugammadex encloses the quaternary ammonium structure of rocuronium, it eliminates the antigenicity of the agent, preventing it from further triggering a type 1 IgE-mediated hypersensitivity reaction [2,5,6]. In our case, after sugammadex administration, the patient showed rapid improvement in blood pressure, heart rate, and SpO2 values, giving credence to this theory. In addition, the low-dose epinephrine infusion used to treat the hypotension caused by the reaction was never increased and was able to be titrated off by the end of the case.

It should be noted that while sugammadex can bind rocuronium and prevent its further role in perpetuating an anaphylactic reaction, it cannot stop an ongoing anaphylactic reaction. It is essential that the standard first-line clinical treatments of anaphylaxis, including epinephrine, rapid fluid resuscitation, antihistamines, steroids, and bronchodilators, are all considered and administered [6,7].

## Conclusions

This report described the case of anaphylaxis/anaphylactic shock in a patient after the induction of general anesthesia. As rocuronium was a likely causative agent, sugammadex was administered to bind rocuronium and eliminate it as an ongoing causative agent. Shortly after sugammadex administration, the patient showed improvement in blood pressure, heart rate, and pulse oximetry values and the operative case was allowed to continue with minimal cardiovascular support for the patient. If anaphylaxis secondary to rocuronium is suspected, sugammadex administration should be strongly considered as a useful novel adjunct to the standard clinical management of anaphylaxis.
